# Last of the Eisenmengers: A Tribute

**DOI:** 10.1016/j.ijcchd.2025.100645

**Published:** 2025-12-05

**Authors:** Craig Broberg

**Affiliations:** Adult Congenital Heart Program, Knight Cardiovascular Institute, Oregon Health and Science University, UHN 62, 3181 SW Sam Jackson Pk Rd, Portland, OR, 97221, United States

This year brought the passing of two of the oldest patients with Eisenmenger Syndrome seen in our Adult Congenital Heart Disease (ACHD) Program. David was 65. Margaret was 75. Both were remarkable survivors. Their loss provides an opportunity to reflect on this interesting condition, even as it gradually fades from the medical profession [1]. Doing so provides insight about physiological adaptations to cyanosis, but also about living on borrowed time, being grateful for life each day, and not being defined by one's diagnosis.

These two individuals, though unknown to each other, had many things in common. Both had large ventricular septal defects that could not be repaired in childhood. Even though corrective surgery was emerging in the decades of their births, circumstances were not always suitable for each child in the world to be repaired, and these two were left out. Both had developed pulmonary hypertension, right to left shunting, and cyanosis, the hallmarks of Eisenmenger syndrome.

Both had been told their entire lives that they would not survive. Their parents were warned they would not make their third birthday, then not their tenth, and so on. Both could recall hearing they'd never go to college or have a family. They had become so accustomed to such grim forecasts that they learned to routinely ignore them with a wave of the hand and a smile. But perhaps because of these constant warnings, they both lived grateful for each day, happy with whatever came, radiating an appreciation for life that most struggle to learn.

Visibly cyanotic, they could both tell stories of being in an Emergency Department and having to talk down the triage team's reaction to their dire oxygen saturation (often in the low 70s) saying, “Don't worry, my sats are always like this,” while shooing away the staffer with the intubation tray at the ready.

Both survived on a fragile balance based on compensatory features to allow them to live quasi anaerobically. These include secondary erythrocytosis, arteriolar recruitment and dilation (visibly manifest in their clubbed fingernails), and a shift of their oxygen-hemoglobin desaturation curves, among others.

As regular clinic visitors for most of their lives, each was something of a celebrity. Staff would offer statements of amazement and well wishes each time they came. We would do our best for them. We'd insist on at least 5 min of rest before measuring their saturations. We would check for iron deficiency and gout. We would image them regularly. We counselled against phlebotomy. But we could appreciate that they had largely done better for themselves than the best of western medicine could hope to achieve under the circumstances. In teaching rounds I would often say, “Try to leave them alone.” Both had atrial arrhythmias on occasion, though these seemed more of a nuisance than a threat, despite their physiologic fragility. Both doubtlessly benefited from pulmonary vasodilators emerging in the early part of this century [2].

Both stayed productive as adults. David drove a truck each day. Margaret was a medical assistant for a local clinic. Neither thought they needed to save for retirement. Both had broad smiles and a sly sense of humor. Both married and benefited from enlarging family circles. David had children, whereas Margaret was told not to conceive. Both were active and faithful in religious circles: David played drums at church; Margaret kept finance records for the local parish. One suspects that family and faith were both meaningful contributors to their longevity. Both outlived family members that they were never expected to outlive.

As the years wore on, so did they, but found themselves gradually slowing down and adjusting activities to match their abilities. Near the end, both felt increasingly cold, as the fires of metabolism found less and less oxygen to keep burning. Winters became hard. They became thin. Slowly and gradually, like a campfire dissipating to embers, both died peacefully at home, with their spouses lovingly next to them grateful for their time together.

I attended the memorial services of both, which gave me wonderful glimpses into their non-clinical selves. I saw clearly that, despite the seeming miracles of their lives, neither of them was labelled by their illness in the real world. Tributes included stories of hobbies or fun vacations, humorous anecdotes, evidence of devoted faith, and appreciation for deep and lasting friendships with those gathered to memorialize them. I kept wondering when the Eisenmenger part of their stories would be highlighted, but it never was. Eulogizers hardly mentioned heart disease at all, other than alluding to an occasional trip to the doctor, without specifics.

I was surprised, but I should not have been. Eisenmenger syndrome was what they had, not who they were. It was apparent that in their personal lives, the medical condition that I found remarkable didn't really matter. Disease did not define them. It is perhaps a lesson all providers should remember for those with chronic illness.

I was first introduced in detail to the Eisenmenger condition during fellowship with mentor Dr. Michael Gatzoulis. We studied a series of such patients, appropriately, on the Paul Wood ward, named for the man who published the seminal paper explaining the physiology of the condition that Viktor Eisenmenger first described [3]. Still, though all patients exhibited the Eisenmenger physiology as Wood defined, each was unique. Each had a story to tell, each lived as best they could, and each seemed grateful for every day knowing that they were living on borrowed time.

Margaret and David are survived by a few other Eisenmenger patients still seen across the world. The oldest in our clinic's diminishing cyanotic cohort is a man with unrepaired truncus arteriosus. He will turn 73 this year, a jaw-dropping milestone. There's no telling if he is the oldest truncus survivor ever, but no doubt he is an extreme outlier statistically. He, too, can tell his story of being given a life he was never meant to have. He became a successful accountant and joined his father in running a busy tax practice. Having outlived his mother, he and his father finally decided they would both retire last year. They had a lift installed in the house, not because of his Eisenmenger's, but so his 90-year-old father wouldn't have to battle the stairs. The days just keep coming one after another, as they do for us all, but he understands that each one is another miracle, another gift.

In citing the successes of pediatric cardiology care over the decades, we insinuate that fewer and fewer Eisenmenger patients like these will be found. Perhaps someday they will be gone. I will treasure the privilege it has been to know and learn from them, and I will miss them.

## Declaration of competing interest

The author declares that he has no known competing financial interests or personal relationships that could have appeared to influence the work reported in this paper.

Thanks to Susan Davidson RN, Victor Menashe MD, and George Pantely MD for their input.
